# Occupational Neurobrucellosis Mimicking a Brain Tumor: A Case Report and Review of the Literature

**DOI:** 10.1155/2017/1434051

**Published:** 2017-02-16

**Authors:** Hussein Algahtani, Bader Shirah, Dina Abdulghani, Roiya Farhan, Raghad Algahtani

**Affiliations:** ^1^King Abdulaziz Medical City and King Saud bin Abdulaziz University for Health Sciences, Jeddah, Saudi Arabia; ^2^King Abdullah International Medical Research Center and King Saud bin Abdulaziz University for Health Sciences, Jeddah, Saudi Arabia; ^3^University of Dammam, Dammam, Saudi Arabia; ^4^King Abdulaziz University, Jeddah, Saudi Arabia; ^5^King Saud bin Abdulaziz University for Health Sciences, Jeddah, Saudi Arabia

## Abstract

Brucellosis is a zoonotic bacterial infection which is transmitted to humans from infected animals and is endemic in many parts of the world including Saudi Arabia. In this article, we report a case of occupational neurobrucellosis that presented with a space-occupying lesion mimicking a brain tumor. We stress on the importance of obtaining detailed social history including occupation to reach the diagnosis in several conditions including brucellosis. We also stress on taking universal precautions when handling any specimens. It may be advisable that manipulation of all unknown specimens arriving at the laboratory should occur in biological safety cabinet until a highly infectious organism is ruled out. Neurobrucellosis should be included in the differential diagnosis in patients presenting with solitary mass lesion mimicking brain tumor especially in endemic areas or high occupational risk group.

## 1. Introduction

Brucellosis is a zoonotic bacterial infection which is transmitted to humans from infected animals and is endemic in many parts of the world including Saudi Arabia. It is a multisystem disease that may present with a broad spectrum of clinical manifestations including undulant fever and musculoskeletal symptoms and signs [1].* Brucella* was first identified from autopsy material of a patient who died on the island of Malta in 1887 by David Bruce. Nine years later, neurobrucellosis was first reported by Matthew Hughes [2]. The heterogeneous clinical presentation of neurobrucellosis may lead to a delay in achieving a proper diagnosis with subsequent development of serious complications. In this article, we report a case of occupational neurobrucellosis that presented with a space-occupying lesion mimicking a brain tumor.

## 2. Case Report

**Figure 1:**
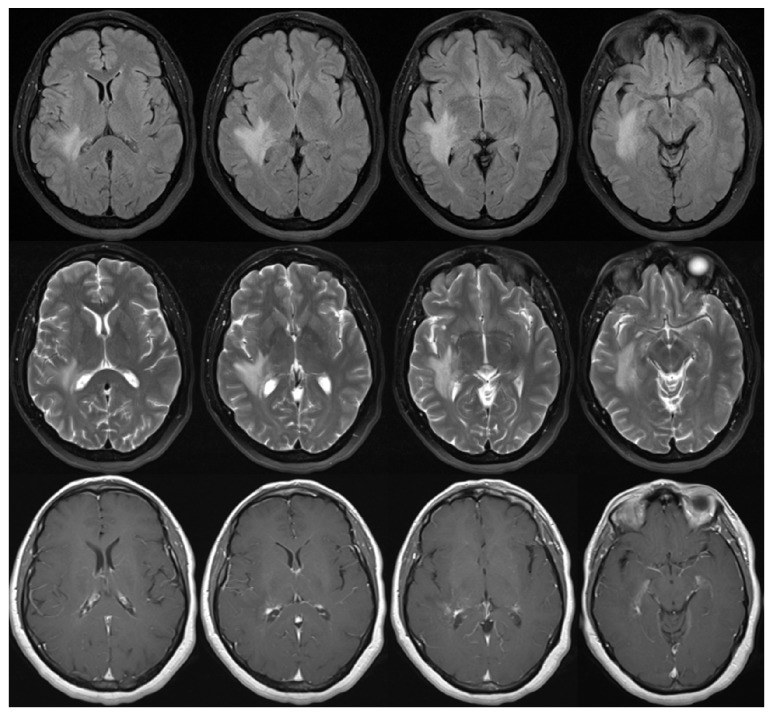
MRI of the brain showing peritrigonal (temporal lobe) deep white matter mass measuring 2.6 × 3.5 cm with surrounding vasogenic edema. There was no restricted diffusion with slightly increased flow at the affected area and normal cerebral blood volume on MR perfusion. Axial and sagittal T1 postgadolinium studies showed minimal enhancement.

**Figure 2:**
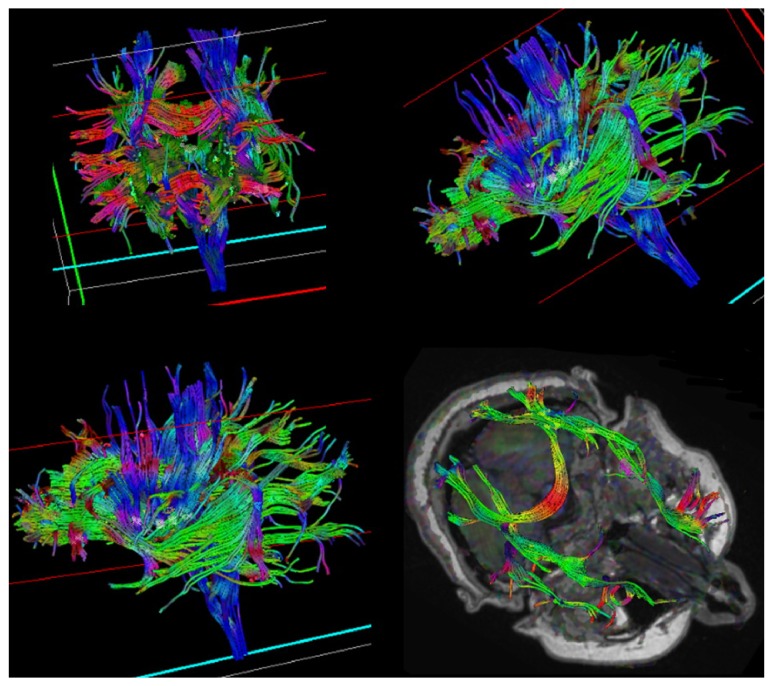
DTI images showing a slight reduction in anisotropy and diminished color brightness at the affected region with a normal organization of the fiber tract.

**Figure 3:**
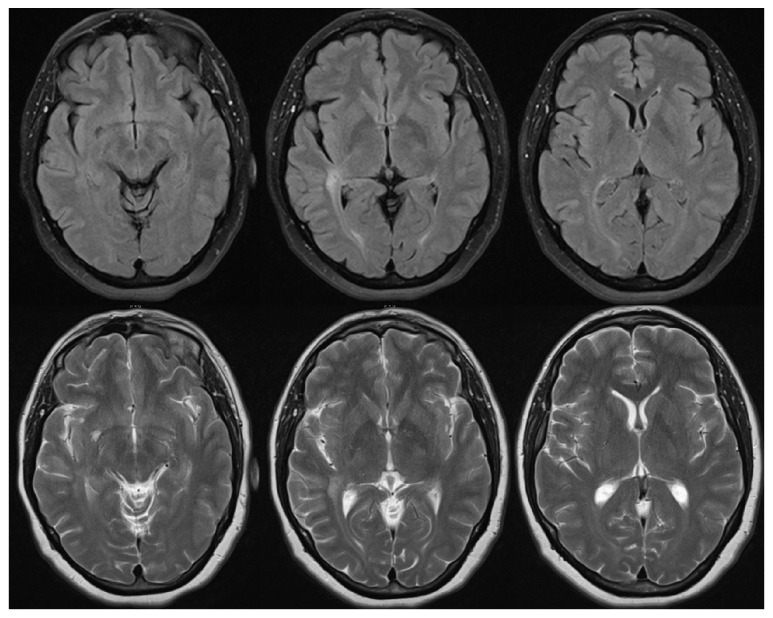
MRI of the brain showing almost complete resolution of the previously noted changes.

A 52-year-old female patient, who works as a microbiologist, presented with a headache, dizziness, and partial seizures. The onset of symptoms was subacute with gradual progression. She was previously healthy with no past medical or surgical diseases or events, and she was not using any medications. There was no history of blood transfusion, raw milk ingestion, tick bites, or drug abuse. She was married with three children. Physical examination showed normal higher mental functions including speech. Cranial nerve examination was unremarkable apart from mild papilledema. Her tendon reflexes were symmetrical and normal with downgoing toes. The rest of her neurological and systemic examination was normal including vital signs. Basic hematological workup including complete blood count, liver function test, erythrocyte sedimentation rate, C-reactive protein, and connective tissue screen were all unremarkable. Other unremarkable important tests included syphilis serology, HIV test, mammogram, and tumor markers. Serum* Brucella melitensis* immunoglobulins were both high measuring IgM 12.2 U/ml and IgG 127.4 U/ml (normal range <12 U/ml). Using enzyme-linked immunosorbent assay (ELISA) for cerebrospinal fluid (CSF),* Brucella* titer was high at 1 : 40 (normal <1 : 20). CSF analysis showed lymphocytic pleocytosis at 63 cells and 88% lymphocytes. Protein was slightly increased, but glucose was normal. Her bacterial culture was negative for both aerobic and anaerobic organisms. Polymerase chain reaction (PCR) for tuberculosis, herpes simplex virus, Epstein–Barr virus, and cytomegalovirus was negative. Both CSF* Brucella* total antibodies and oligoclonal bands were positive with five well defined gamma restriction bands which were not present in the corresponding serum sample. Magnetic resonance imaging (MRI) of the brain revealed peritrigonal (temporal lobe) deep white matter mass measuring 2.6 × 3.5 cm with surrounding vasogenic edema. There was no restricted diffusion with slightly increased flow at the affected area and normal cerebral blood volume on MR perfusion. Axial and sagittal T1 postgadolinium studies showed minimal enhancement (Figure 1). Diffusion tensor imaging (DTI) revealed a slight reduction in anisotropy and diminished color brightness at the affected region with a normal organization of the fiber tract (Figure 2). MR spectroscopy was suggestive of inflammatory rather than a neoplastic process. The radiological deferential diagnosis included neurosarcoidosis, lymphoma, and low-grade glioma. CSF cytology and flow cytometry were normal. Given the clinical, laboratory, and radiological features, the patient was diagnosed with neurobrucellosis and was started on rifampicin (600 mg/day), doxycycline (100 mg twice a day), and sulfamethoxazole-trimethoprim (960 mg twice a day). The patient's symptoms resolved gradually with concomitant improvement in her MRI images. She was treated for a period of 45 days with clinical but no radiological improvement. CSF analysis was repeated which showed improvement of cell count and protein concentration, but the values were not normalized yet. We instructed the patient to continue using the medications for a minimum of 6 months. The CSF analysis was repeated and completely normalized, and her MRI showed almost complete resolution of the previously noted changes. She remained symptom-free until now (Figure 3).

## 3. Discussion

Brucellosis is caused by organisms from the bacterial genus* Brucella*, which are gram-negative intracellular aerobic rods. Genus* Brucella* consists of eleven species with the most commonly isolated being* Brucella melitensis*,* Brucella abortus*,* Brucella suis*,* Brucella ovis*, and* Brucella canis*. Humans can acquire the infection through ingestion of unpasteurized milk or milk products from infected animals, inhalation of aerosols, and skin abrasions by direct contact with the genital mucosa of infected animals when helping the females to abort or the contact with their secretions. The organism can survive for up to two months in soft cheese locally made from goats or sheep's milk. In addition, it can survive for at least six weeks in dry contaminated soil and six months in damp soil or liquid manure kept under cool dark conditions. Brucellosis remains the most common bacterial zoonosis worldwide. It is a common disease in the Arabian Peninsula and the countries bordering the Mediterranean Sea [3].

*Brucella* is a well-known health hazard to laboratory workers who handle cultures or infected samples with an estimated incidence of laboratory-associated infection in 2% of the reported cases. The most common causative agent isolated is* Brucella melitensis* (81% of reported laboratory-associated infection) followed by* abortus* and* suis*. Laboratory infection could be due to accidents, direct contact, inoculation through needle-stick injuries, and contamination of skin and mucous membranes through spills or splashes into eyes, mouth, or nose. Many of the exposures were caused by handling specimens where brucellosis was not suspected clinically [4].

Communication between clinicians and laboratory workers is important to help the laboratory staff take extra precautions during the identification of a specimen. It may be advisable that manipulation of all unknown specimens arriving at the laboratory should occur in biological safety cabinet until a highly infectious organism is ruled out [4]. In our patient, she was handling different types of specimens including blood, tissue, urine, and so on, which made her susceptible to the infection. There was no other risk factors including raw milk or other dairy products ingestion. In a study conducted by Traxler et al. [4], microbiologists were found to be the most frequently exposed group of laboratory workers followed by researchers and clinicians.

Brucellosis is a systemic disease that may involve almost every organ system. The exact mechanism by which the organism reaches the nervous system is still unclear. Involvement of the central nervous system (CNS) has only been detected in 3–5% of patients. Once bacteremia occurs, the organism travels to the meninges producing polyradiculoneuropathy, meningitis, or meningoencephalitis. One of the rare consequences of the direct deleterious effect of the organism invading the CNS is the occurrence of a mass lesion or brain abscess which can be documented radiologically and pathologically. Another possible mechanism is an immune-mediated damage of nervous tissue due to the release of circulating endotoxins or to the immunological and inflammatory reaction of the host to the presence of these organisms within the nervous system or other tissues of the body [5].

Clinical features of neurobrucellosis include back pain, areflexia, paraparesis, cranial nerve involvement, myelitis, and meningovascular involvement including strokes, neuropathy, or depression. The vestibulocochlear nerve has been reported to be the most commonly affected cranial nerve where the patients present with vestibuloacoustic neuritis or hearing loss [6].

Laboratory tests conducted for the diagnosis of neurobrucellosis include blood and CSF cultures, serum and CSF agglutination tests, and ELISA. Examination of the CSF typically reveals an elevated protein concentration, a normal or slightly depressed glucose concentration, and a moderate lymphocytic pleocytosis. Although positive blood and CSF cultures are the gold standard for diagnosis, the low rate of* Brucella* isolation from the CSF (<20%) and the long time needed for the results had made it suboptimal. Therefore, the diagnosis usually relies on the detection of antibodies to* Brucella* lipopolysaccharide (LPS) in CSF by agglutination tests (positive Wright's agglutination or Coombs' test at C1:160 titers) or ELISA. Although brain biopsy is the gold standard investigation to diagnose several conditions, clinical examination and paraclinical tests including serology might score the diagnosis and avoid performing this invasive procedure. Imaging findings can range from normal imaging to inflammatory changes (granulomas, abnormal enhancement of the meninges, perivascular space, or lumbar nerve roots), white matter, or vascular changes [7].

To date, neurobrucellosis presenting with a space-occupying mass mimicking a cerebral tumor has been documented in only three patients [8–10]. Bacterial isolation was possible in only one patient, while in the other two cases, the diagnosis was suggested by brain biopsy. Our case is the first case where the diagnosis was made based on blood and CSF serology. Neurobrucellosis should be included in the differential diagnosis in patients presenting with solitary mass lesion mimicking brain tumor especially in endemic areas or high occupational risk group.

The treatment of neurobrucellosis is still controversial, and no consensus regarding the best treatment has been established. The primary drugs of choice due to their enhanced CNS penetrance, tolerability, and high gastrointestinal absorption are doxycycline, rifampicin, sulfamethoxazole-trimethoprim, ciprofloxacin, and ceftriaxone. In addition to neurotoxicity, streptomycin has low CSF penetrance and has been accused of being inappropriate. Treatment duration in neurobrucellosis is suggested to be several months (a minimum of 6–8 weeks) depending on the patient's response and should generally be continued until the CSF analysis has returned to normal and the MRI abnormalities disappear [11].

## 4. Conclusion

We stress on the importance of obtaining detailed social history including occupation to reach the diagnosis in several conditions including brucellosis. We also stress on taking universal precautions when handling any specimens. It may be advisable that manipulation of all unknown specimens arriving at the laboratory should occur in biological safety cabinet until a highly infectious organism is ruled out. Neurobrucellosis should be included in the differential diagnosis in patients presenting with solitary mass lesion mimicking brain tumor especially in endemic areas or high occupational risk group.
